# A first, naturally occurring substitution at the second pyrethroid receptor of voltage‐gated sodium channel of *Aedes aegypti*

**DOI:** 10.1002/ps.6324

**Published:** 2021-02-25

**Authors:** Kentaro Itokawa, Shogo Furutani, Aki Takaoka, Yoshihide Maekawa, Kyoko Sawabe, Osamu Komagata, Takashi Tomita, José Luiz de Lima Filho, Luiz Carlos Alves, Shinji Kasai

**Affiliations:** ^1^ Pathogen Genomics Center National Institute of Infectious Diseases Tokyo Japan; ^2^ Department of Medical Entomology National Institute of Infectious Diseases Tokyo Japan; ^3^ Laboratório de Imunopatologia Keizo Asami Universidade Federal de Pernambuco Recife Brazil; ^4^ Instituto Aggeu Magalhães‐FIOCRUZ/PE Recife Brazil

**Keywords:** pyrethroids, knockdown resistance, *Aedes aegypti*, sodium channel

## Abstract

**BACKGROUND:**

*Aedes aegypti* is a remarkably effective mosquito vector of epidemiologically important arboviral diseases including dengue fever, yellow fever and Zika. The present spread of resistance against pyrethroids, the primary insecticides used for mosquito control, in global populations of this species is of great concern. The voltage‐gated sodium channel (VGSC) in the nervous system is the known target site of pyrethroids in insects. Past studies have revealed several amino‐acid substitutions in this channel that confer pyrethroid resistance, which are known as knockdown resistance (*kdr*) mutations.

**RESULTS:**

This study investigated a laboratory colony of *Ae. aegypti*, MCNaeg, established from larvae collected in Rio de Janeiro, Brazil in 2016. The MCNaeg colony showed strong resistance against pyrethroids without laboratory selection. Of the two *VGSC* gene haplotypes present within this colony, one harbored three known *kdr* mutations, V410L, V1016I, and F1534C, and the other harbored only the known F1534C mutation. In latter haplotype, we also found novel amino‐acid substations including V253F. Previous molecular modeling and electrophysiological studies suggest that this residue serves a pyrethroid‐sensing site in the second receptor, PyR2. Our genetical analysis showed that the haplotype harboring V253F and F1534C is associated with equal or slightly stronger resistance than the other triple *kdr* haplotype to both Type I and Type II pyrethroids.

**CONCLUSION:**

The novel substitution V253F is potentially involved in pyrethroid resistance in *Ae. aegypti*. Further studies are needed to elucidate the role of this substitution in the pyrethroid susceptibility of VGSC.

© 2021 The Authors. *Pest Management Science* published by John Wiley & Sons Ltd on behalf of Society of Chemical Industry.

## Graphical abstract

We found a novel amino‐acid substitution V253F in the voltage‐gated sodium channel in *Aedes aegypti* which is potentially involved in pyrethroid resistance of this vector mosquito

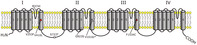

## 1 INTRODUCTION

Pyrethroids include a number of natural and synthetic chemicals used as the active ingredients of insecticides. Because of their rapid effect and highly selective toxicity to insects *versus* mammals, pyrethroids are essential for the control of medically important insect vectors. They are used in indoor residual spraying formulations and insecticide‐treated bed net. The insecticidal activity of pyrethroids results from their inhibition of voltage‐gated sodium channel (VGSC) in the nervous system. Pyrethroids bind to the insect VGSC and prolong the channel's open state, hereby prohibiting normal signal transduction and causing paralysis.^1^ Several specific amino‐acid substitutions in the VGSC are known to decrease its sensitivity to pyrethroids.^2^, ^3^ These amino‐acid substitutions, which are known as genetic factor *kdr* (*knockdown resistance*), has been observed in many agricultural and medically important arthropod pests.^2^, ^3^

The alpha subunit of eukaryotic VGSC consists of a single polypeptide chain including four homologous repeat domains (I–IV), each having six transmembrane segments (S1–S6). Recent molecular modeling and electrophysiological studies^4^, ^5^ suggest the presence of two pyrethroid receptors, PyR1 and PyR2, within the VGSC. In the proposed model, each receptor includes the residues located in IIL45 (the loop between the transmembrane segments S4 and S5 in domain II), IIS5 (the transmembrane segment S5 in domain II), IIS6, and IIIS6 and in IL45, IS5, IS6, and IIS6, respectively, with rotationally quasi‐symmetric disposition. It is considered that simultaneous binding of pyrethroid molecules to each receptor is required for inhibition of VGSC. Many of the residues located in the two receptors correspond known *kdr* substitutions associated with pyrethroid resistance in nature.^5^

*Aedes aegypti*, the yellow fever mosquito, is a remarkably effective vector for numerous important human arbovirus diseases, including dengue fever, yellow fever, Chikungunya, and Zika. In this species, the amino‐acid variation associated with pyrethroid resistance include V410L, G923V, L982W, S989P, A1007G, I1011V/M, V1016G/I, T1520I, F1534C/L, and D1763Y (amino‐acid positions corresponding to the *Musca domestica* VGSC model) (see references in Fan *et al*.,^6^ Du *et al*.,^7^ and Chen *et al*.^8^). In Latin America, F1534C is the *kdr* substitution most frequently reported to date. V1016I (V1023I in some studies) is another *kdr* variant common in Latin America, along with the F1534C substitution.^9^ A congenic strain that inherited the F1534C haplotype from Thailand in the susceptible strain (ROCK) genetic background exhibited seven‐fold and 16‐fold resistance against permethrin (a type I pyrethroid) and deltamethrin (a type II pyrethroid), respectively.^10^ The contribution of F1534C on the deltamethrin resistance phenotype, however, is still debatable. Multiple electrophysiological studies on VGSC heterologously expressed in *Xenopus* oocytes have shown that F1534C alone confers slight resistance to permethrin and dichlorodiphenyltrichloroethane (DDT) but not to deltamethrin.^11^, ^12^, ^13^ Interestingly, V1016I enhances the effect of F1534C on the permethrin resistance in the electrophysiological experiment; this V1016I + F1534C double mutant even confers resistance to deltamethrin even though neither of the two mutations alone confers notable deltamethrin resistance.^11^ The V410L variant, which often associates with F1534C, was first found in Brazil.^13^ An electrophysiological study^13^ of encoded channel indicates that this mutation confers strong resistant to both permethrin and deltamethrin. The channel with the double mutation V410L + F1534C is more resistant to permethrin than channels with either of the mutation alone. Concerningly, several reports show that a haplotype harboring all three mutations (V410L + V1016I + F1534C) already exists in natural population in America^6^, ^14^, ^15^, ^16^ and more recently in Africa.^17^

In this article, we report several novel substitutions found in the *Ae. aegypti* VGSC from a pyrethroid‐resistant colony collected in Brazil. Among those substitutions, V253F resided at the residue V^1k11^, which has been implicated as one of the pyrethroid‐sensing residues in PyR2 under the dual‐receptor model.^4^ The results obtained in this study prompt further electrophysiological and population genetical characterizations of this mutation to evaluate potential impact for future control of *Ae. aegypti*.

## 2 MATERIALS AND METHODS

### 2.1 Insects

Twelve *Aedes aegypti* larvae were collected on March 13, 2016 at Maracanã, Rio de Janeiro, Brazil. The larvae were kept in the laboratory insectarium and finally emerged into five males and seven females. Random crossing of those adult mosquitoes founded the MCNaeg colony. The SMK strain, which was originally from the United States and has been maintained in the laboratory for at least 20 years without exposure to insecticides,^18^ was used as susceptible control. Larvae were fed insect foods purchased from the Oriental Yeast Company (Tokyo, Japan) and adults were fed 2% sugar water. Female mosquitoes were fed blood from a live mouse (Slc:ICR). The environmental condition of the insectarium was set on 25 °C under 16 h light/8 h dark photoperiodic cycle. Animal care and protocols were approved by the Animal Ethics Committee of National Institute of Infectious Diseases, Japan (approval number 119051).

### 2.2 Chemicals

Two pyrethroids were used for testing insecticide susceptibility: Permethrin (91.2%) was obtained from Sumitomo Chemical (Tokyo, Japan) and deltamethrin (99.4%) was obtained from GL Sciences (Tokyo, Japan). Piperonyl butoxide (PBO, 98.0%) was obtained from FUJIFILM Wako Pure Chemical Co. (Osaka, Japan).

### 2.3 Adult bioassay

Bioassays were conducted to assess the pyrethroid resistance of adult mosquitoes (mated females at the second inbred generation) by topical application as described previously.^18^ Mosquitoes unable to stand at the bottom of the cup 24 h after treatment were considered dead. For the original MCNaeg colony, four batches of 20 females (total 80 females) were treated with 5.87 and 58.7 ng of permethrin. To estimate the half lethal dose (LD_50_), at least five different doses of permethrin or deltamethrin were used to achieve mortality > 0% and < 100%. At least 40 mosquitoes were used per single dose. To estimate the contribution of cytochrome P450s to resistance, 2 μg PBO (in 0.22 μL acetone) was applied to the thoracic notum of the mosquitoes 2 h before pyrethroid application. LD_50_ values for each population were calculated by fitting numbers of dead and surviving mosquitoes to log‐probit model^19^ using the *glm* function in R v3.^20^

### 2.4 Association between *kdr* genotypes and permethrin susceptibility

For the polymerase chain reaction (PCR) template, genomic DNA (gDNA) from dead and surviving mosquitoes of the MCNaeg colony after the exposure to 58.7 ng permethrin was prepared from two hind legs using the alkaline lysis method^21^ modified for mosquito legs.^22^ Domains I, II, and III of the *VGSC* genes were partially amplified and directly sequenced as previously described.^23^ For genotyping the domain I V410 residue, primers Ae410F1 (5′‐TTACGATCAGCTGGACCGTG‐3′) and Ae434R3 (5′‐CTTCTTCCTCGGCGGCCTC‐3′) were used for PCR amplification. The cycling conditions for PCR were as follows: initial denaturation at 95 °C for 2 min, followed by 35 cycles of 98 °C for 10 s, 55 °C for 30 s, 68 °C for 30 s, and a final extension step at 68 °C for 5 min. The 180 bp amplicon was directly sequenced with primer Ae410F2 (5′‐ATCAGCTGGACCGTGGCA‐3′) and genotyped from the electropherogram. Fisher's exact test was conducted for difference in allele frequency between survived and dead group using the *fisher. test* function in R v3.

### 2.5 Isolation of two sub‐colonies from the MCNaeg colony

Two distinct haplotypes were identified in the MCNaeg colony that harbored the V410 and L410 alleles. To separate these haplotypes into two sub‐colonies, pupae of the MCNaeg colony were isolated, and each emerged adult was genotyped by PCR and direct sequencing using a hind leg as described earlier. Individuals with a homozygous genotype for either allele were selected and crossed separately. Isolated sub‐colonies were designated as MCNaeg‐C (with V410) or MCNaeg‐LIC (with L410).

### 2.6 Targeted capture sequencing

Genomic DNA was extracted from four individual pupae in each sub‐colony using MagExtractor ‐Genome‐ (Toyobo, Japan) as described previously.^16^ Illumina library construction and hybridization capture was conducted with the biotinylated oligo probe designed from the *Ae. albopictus* VGSC gene, whose exons show > 92.5% homologies to corresponding exons in *Ae. agypti* except one tiny optional exon 16.5.^16^ The libraries were pooled along with libraries for other projects and sequenced for 150 bp at both ends in MiniSeq (Illumina, Inc., Foster City, CA, USA) with the 300‐cycle Mid‐Output Sequencing kit. A range of 188 481 to 240 793 read pairs (50–72 Mb) was obtained per individual. The row next generation sequencing (NGS) reads were deposited in the DNA Data Bank of Japan (DDBJ) Sequence Read Archive (DRA) under accession number DRR234414–DRR234421.

### 2.7 Bioinformatic analysis

The obtained NGS read data were used to genotype and functionally annotate variants with respect to *VGSC* gene coding sequence (CDS) using the automated pipeline MoNaS (https://github.com/ItokawaK/MoNaS),^16^ which depends on BWA,^24^ SAMTools,^25^ BEDTools,^26^ and FreeBayes.^27^ SKESA v.2.3.0^28^ with default parameters (minimal kmer length = 21, maximum noise‐to‐signal ratio = 0.1) was used to assemble the NGS reads obtained from the targeted capture sequencing. The contigs containing the *VGSC* exons and flanking introns for each sub‐colony were submitted to DDBJ under accession number LC557523–LC557562.

## 3 RESULTS

### 3.1 Pyrethroid susceptibility and two VGSC haplotypes in MCNaeg

After permethrin exposure of 5.87 and 58.7 ng per mosquito doses, the females of the MCNaeg colony exhibited mortality rates of only 10.0% (*n* = 80) and 28.8% (*n* = 80), respectively. Because these doses were expected to eliminate almost 100% susceptible *Ae. aegypti*,^18^ and corresponded to 99% lethal dose (LD_99_) and its ten times equivalent, respectively, for the susceptible strain of *Ae. albopictus* (the HKM strain),^23^ the MCNaeg colony was considered highly resistance against permethrin. All 80 females used for the 58.7 ng permethrin challenge (56 survivors and 24 dead) were genotyped by direct sequencing of three regions of the *VGSC* gene that include codons of known *kdr* variations (V410L, V1016G/I, and F1534C). All of these mosquitoes were homozygous for the C1534 allele, while segregations of genotypes at the residue 410 (V or L) and 1016 (V or I) were observed. Because there were only three combinations of genotypes at the two segregating sites among the 80 females (Table 1), we suspected the MCNaeg colony had only two *VGSC* haplotypes, V–V–C or L–I–C corresponding to the residues 410, 1016, and 1534. Frequency of the L–I–C haplotype among dead and surviving mosquitoes was 70.8% and 37.5%, respectively. Intriguingly, the V–V–C haplotype seemed to have a slightly higher chance of being selected over the L–I–C haplotype under this permethrin exposure (Table 1, *P* < 0.05), even though the V–V–C haplotype had only F1534C as a ‘known’ *kdr* mutation.

**Table 1:** Association between voltage‐gated sodium channel (VGSC) genotypes and responses to 58.7 ng permethrin challenge in the MCNaeg colony

Genotype			Dead	Survived	Mortality (95% confidence interval)
V410L	V1016I	F1534C	*n* = 24	*n* = 56	
V/V	V/V	C/C	1	4	0.20 (0.0051–0.72)
V/L	V/I	C/C	6	31	0.16 (0.062–0.32)
L/L	I/I	C/C	17	21	0.45 (0.29–0.62)
Frequency of the V‐V‐C allele^a^ (95% confidence interval)			0.17 (0.075–0.30)	0.35 (0.26–0.44)	

^*a*^ Significant difference in the allele frequency between dead and survived (*P* = 0.023) in Fisher's exact test.

### 3.2 Isolation of two sub‐colonies with fixed VGSC haplotypes

**Figure 1:**
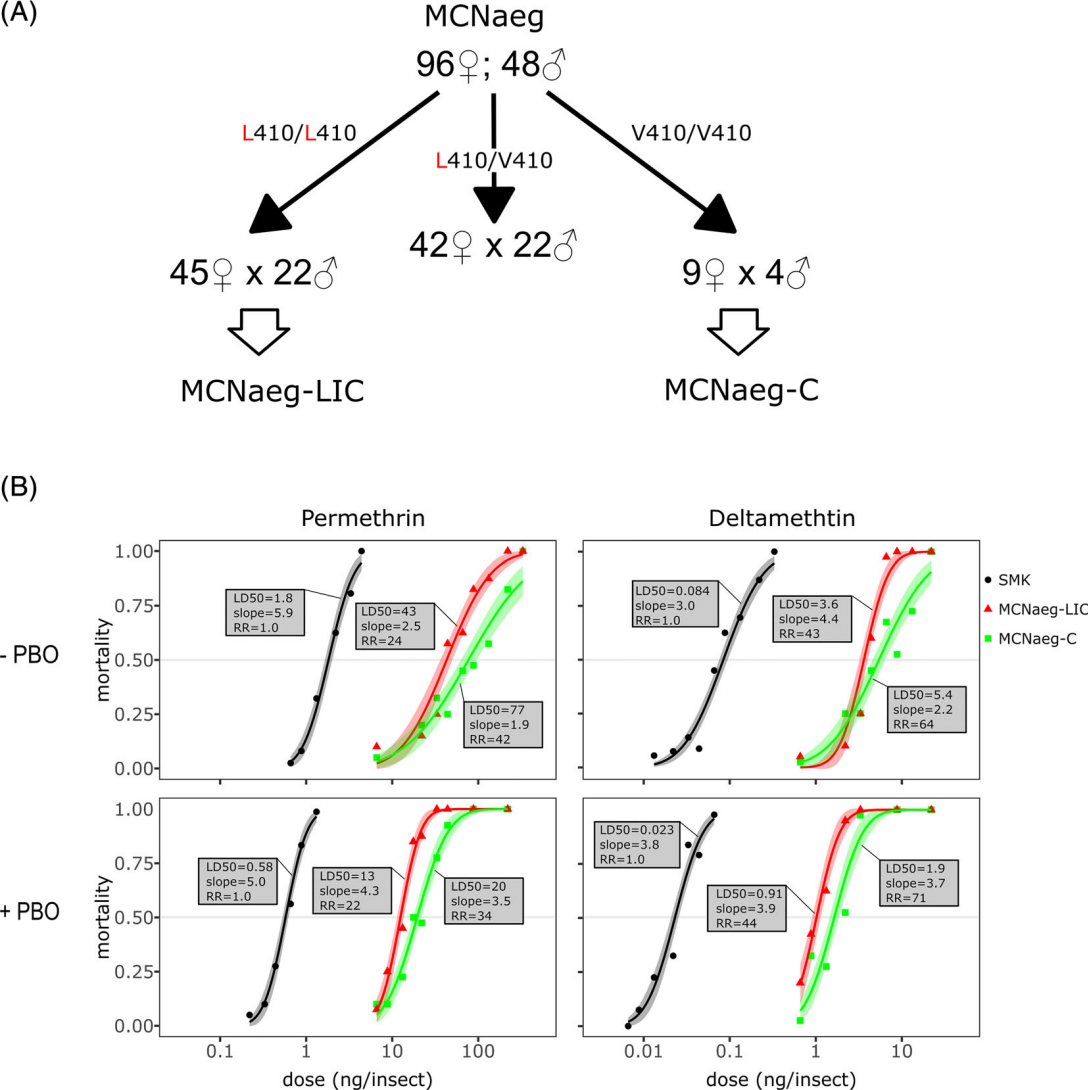
(A) Schematic diagram depicting the selection of two sub‐colonies MCNaeg‐LIC and MCNaeg‐C from the MCNaeg colony with the V410L genotype. (B) Dose‐response curve for permethrin and deltamethrin either with (+) or without (−) PBO for MCNaeg‐LIC, MCNaeg‐C and susceptible (SMK) adult females. Lines represent fitted curves for the log‐probit model, and bands with translucent colors are 95% confidential interval for morality. The LD_50_, slope and RR of each fitted curve are shown in the box.

We isolated 144 unmated mosquitoes (96 females; 48 males) in the MCNaeg colony and genotyped them at V410L. Frequencies of each genotype (V/V, V/L, and L/L) were 9, 42, and 45, respectively, among female and 4, 22, and 22, respectively, among males. The L410 homozygous mosquitoes (45 females; 22 males) were separated from the V410 homozygous mosquitoes (nine females; four males) kept in separate cages, and mated. This selection by genotype resulted in the isolation of two sub‐colonies, MCNaeg‐LIC (fixed with respect to L410) and MCNaeg‐C (fixed with respect to V410) (Fig. 1(A)). As expected from the results of phenotype–genotype associations in the original MCNaeg colony (Table 1), the MCNaeg‐C sub‐colony had equal or slightly higher resistance for both permethrin and deltamethrin (resistance ratio, RR, compared to the SMK strain were 42‐ and 64‐fold, respectively) than did the MCNaeg‐LIC (24‐ and 43‐fold) (Fig. 1(B)). Treatment with PBO, an inhibiter of cytochrome P450 detoxification enzymes that may be involved in pyrethroid resistance^18^ along with the *kdr* mechanism, did not dramatically reduce the resistance ratios both in MCNaeg‐LIC (22‐ and 44‐fold) and MCNaeg‐C (34‐ and 71‐fold) (Fig. 1(B)).

### 3.3 Sequencing the VGSC coding sequence

**Figure 2:**
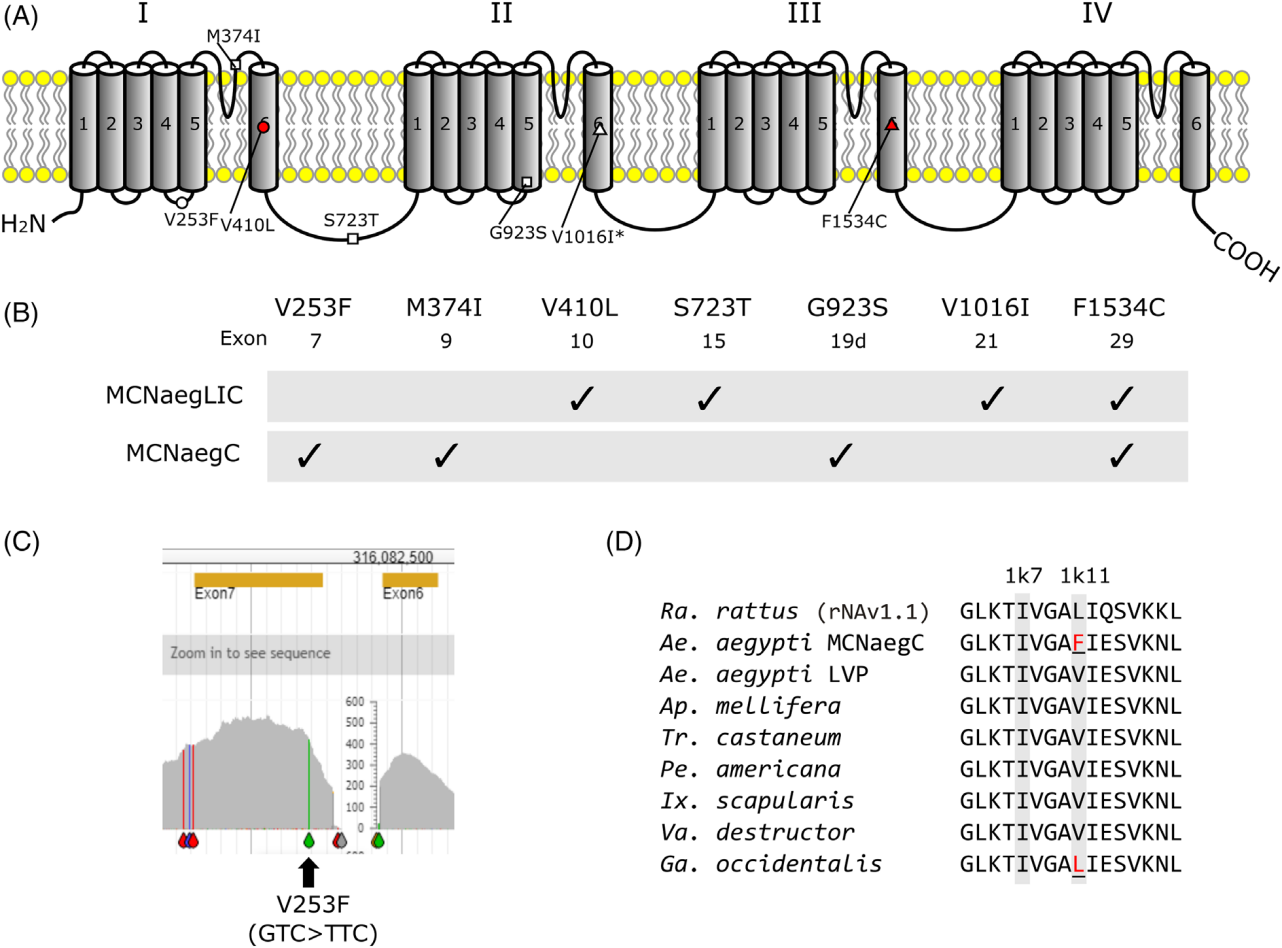
(A) Mapping of the amino‐acid substitutions discovered in haplotypes of the MCNaeg‐LIC and MCNaeg‐C sub‐colonies to the VGSC protein. Triangle and circles points indicate residues consisting of pyrethroid receptors 1 and 2, respectively, according to the dual‐receptor model by Du *et al*.^4^, ^5^ Rectangle points indicate residues with no known association with insecticide resistance. Filled points indicate known resistance substitutions with evidence from electrophysiological studies. V1016I annotated by an asterisk has been shown to enhance pyrethroid insensitivity mediated by F1534C.^11^ (B) Amino‐acid substitutions in each *VGSC* haplotype. The exon in which each substituting mutation resides is indicated. (C) NGS read mapping view for a MCNaeg‐C individual in exon 7 of *VGSC* (chromosome III), including the V253F substation. (D) An alignment of the IL45 linker region of the sodium channel in the rat *Rattus rattus* rNAv1.1 (NP_110502), and *Ae. aegypti* Liverpool (LVP) and MCNaeg‐C (LC557527) strains, the bee *Apis mellifera* (XP_006561583.1), the beetle *Tribolium castaneum* (XP_015837360.1), the cockroach *Periplaneta americana* (ACX44801.1), the tick *Ixodes scapuraris* (EEC03677.1), the mite *Varroa destructor* (XP_022662766.1), and the mite *Galendromus* (*Metaseiulus*) *occidentalis* (XP_028966827.1). Two pyrethroid‐sensing residues, 1 k7 and 1 k11 (according to Du *et al*.^4^), are highlighted.

The entire CDS of the *VGSC* gene was sequenced using targeted capture sequencing with hybridization probes designed from the *Ae. albopictus VGSC* gene.^16^ Four individuals were sampled from each sub‐colony for sequencing analysis. In addition to the known variants V410L, V1016I, and F1534C, we identified four other amino‐acid substitutions: V253F (GTC > TTC at exon 7), M374I (ATG > ATA at exon 9), S723T (TCT > ACT at exon 15), and G923S (GGT > AGT at a mutually exclusive exon 19d) (Fig. 2(A)). The haplotype in the MCNaeg‐LIC sub‐colony appeared to harbor amino‐acid substitutions V410L, S723T, V1016I, and F1534C. The haplotype in the MCNaeg‐C sub‐colony, however, harbored amino‐acid substitutions V253F, M374I, G923S, and F1534C (Fig. 2(B, C)).

**Figure 3:**
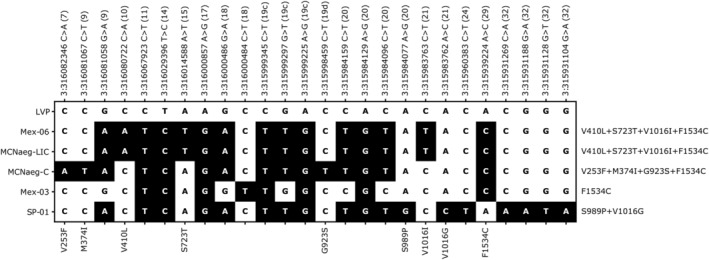
All synonymous and non‐synonymous nucleotide substitutions found in entire CDSs of *VGSC* genes in the Liverpool strain (genome reference, AaegL5), MCNaeg‐LIC and MCNaeg‐C sub‐colonies in this study, and specimens Mex‐03, Mex‐06, and SP‐01 from our previous study (SRA accession: DRR167899, DRR167902, and DRR167913, respectively),^16^ as shown at the left side of the table. Genomic coordination of each substitution in the AaegL5 assembly are shown at the upper side of the table. Characters in parenthesis indicate *VGSC* gene exon numbers in which each nucleotide locus resides. Corresponding amino‐acid substitutions are shown at the bottom of the table if a substitution is non‐synonymous. Haplotypes with respect to amino‐acid substitutions are shown on the right side of the table.

No heterozygous genotype within the CDS was found in any of the individuals sampled from either sub‐colony, allowing us to reconstruct the full sequences for each *VGSC* haplotype by assembling the NGS reads. The complete CDSs of the *VGSC* gene were compared to that of three other haplotypes in two *Ae. aegypti* laboratory colonies; SP‐01 from a colony established from mosquitoes collected in Singapore 2009, and Mex‐03 and Mex‐06 from colony established from mosquitoes collected in Monterrey, Mexico 2008^16^ (Fig. 3). A total of 26 synonymous and non‐synonymous polymorphic sites were present within the whole CDS (approximately 6 kb) compared to the reference genome assembly the Liverpool strain, AaegL5.^29^ The two haplotypes MCNaeg‐LIC and Mex‐06, which shared identical amino‐acid substitutions (V410L + S723T + V1016I + F1534C)^16^ had exactly the same set of nucleotide substitutions across the entire CDS.

## 4 DISCUSSION AND CONCLUSION

The *Ae. aegypti* colony, MCNaeg, which has been founded from mosquitoes collected in Rio de Janeiro Brazil, showed > 50% survivability on 58 ng/insect dose of permethrin, which was 38‐fold higher dose than LD_50_ of susceptible SMK strain (Fig. 1(B)), without laboratory selection. The colony included two different haplotypes of the *VGSC* gene harboring amino‐acid substitutions V410L + S723T + V1016I + F1534C (MCNaeg‐LIC) and V253F + M374I + G923S + F1534C (MCNaeg‐C) compared to the reference genome assembly (AaegL5).^29^

Saavedra‐Rodriguez *et al*.^15^ found *VGSC* haplotypes harboring the three *kdr* substitutions V410L, V1016I, and F1534C in specimens collected during 2002–2005. The S723T substitution located at the intracellular linker between domains I and II has been reported in at least three studies on *Ae. aegypti* collected in Mexico^16^, ^30^ and Puerto Rico.^31^ In two of these studies,^16^, ^31^ the S723T substitution was associated with V410L, V1016I, and F1534C. Of note, the nucleotide sequence of the MCNaeg‐LIC *VGSC* haplotype is identical to that reported for the entire CDS region of the V410L + S723T + V1016I + F1534C haplotype found in mosquitoes collected in Mexico 2008.^16^ This indicates that these haplotypes share the same evolutionally origin. Thus far, no evidence, yet, suggests that S723T is involved in VGSC to pyrethroids. Electrophysiological study on *Ae. aegypti* VGSC with the triple (V410L + V1016I + F1534C) and quadruple (V410L + S723T + V1016I + F1534C) substitutions are needed to correctly understand the selective advantage of these haplotypes in natural population.

The other haplotype (V253F + M374I + G923S + F1534C), isolated in the MCNaeg‐C sub‐colony, was genetically associated with at least comparable pyrethroid resistance to the V410L + S723T + V1016I + F1534C haplotype even though the F1534C is the only known *kdr* mutation implicated in pyrethroid resistance in the MCNaeg‐C thus far. Other three substitutions, V253F (at IL4), M374I (at IL5), and G923S (at IIS5) are novel in *Ae. aegypti*, and probably also in arthropods. Of these amino‐acid substitutions, we consider V253F to be particularly noteworthy. A valine in this residue is highly conserved among arthropods (Fig. 2(D)). Du *et al*.^4^, ^5^ state that this residue (also indexed as V^1k11^) constitutes the VGSC's second pyrethroid‐receptor, PyR2, in their dual‐receptor model. An electrophysiological study using *Xenopus* oocytes showed that substitution of this valine residue to alanine (V253A) in *Ae. aegypti* VGSC dramatically reduces the sensitivity of the channel to both deltamethrin and permethrin.^4^ The effect of the V253F substitution on the electrophysiological characteristics of the channel is yet unknown. While G923S is a novel substitution, another substitution at the same amino‐acid site, G923V, has been reported in *Ae. aegypti* collected in three Latin American countries.^32^ Although these colonies had pyrethroid resistance phenotypes, it is unclear whether G923V is responsible for the resistance because the I1011M *kdr* substitution was present in the same haplotype.^32^

To the best of our knowledge, no amino‐acid substitution at the V^1k11^ residue has been reported in arthropods in nature. However, our search of the Genbank database (July 2020) identified the L253 VGSC allele in a predatory mite species *Galendromus* (*Metaseiulus*) *occidentalis*, which is used as a commercial biological insecticide, from entries of the genome assembled for a carbaryl‐organophosphate‐sulfur selected resistance inbred strain^33^ (Fig. 2(D)). Intriguingly, leucine at this site is homologous to that of the mammalian VGSC alpha subunits. According to Du *et al*.,^4^ L^1k11^V conversion on one of the rat sodium channels (rNav1.4) significantly increased the channel sensitivity to deltamethrin, indicating that the differing residues at this site may explain the selective action of pyrethroids between mammalian and insect sodium channels.^4^ Thus, the prospect that a V^1k11^L mutation possibly occurred in this mite VGSC (conversion to mammalian type) confers the pyrethroid resistance of this beneficial predator^34^ is intriguing.

We observed the MCNaeg‐C sub‐colony fixed with the V253F + M374I + G923S + F1534C haplotype showed comparable or rather slightly higher resistance to the MCNaeg‐LIC sub‐colony with three *kdr* mutations (V410L, V1016I, and F1534C). At present, however, whether the V253F + M374I + G923S + F1534C haplotype actually confers the same level of resistance as V410L + S723T + V1016I + F1534C is unclear because genetic studies alone cannot exclude the presence of another resistance factor hitchhiking to the *VGSC* haplotype. Further studies, especially electrophysiological and genome editing analyses,^35^ are required to fully understand the contribution of the V253F substitution to the resistance phenotype.

## CONFLICT OF INTEREST

There is no interest to declare with this study.
